# Skin Color Preferences in a Malaysian Chinese Population

**DOI:** 10.3389/fpsyg.2019.01352

**Published:** 2019-06-19

**Authors:** Kok Wei Tan, Ian D. Stephen

**Affiliations:** ^1^ School of Psychology and Clinical Language Sciences, University of Reading Malaysia, Iskandar Puteri, Malaysia; ^2^ School of Psychology, University of Nottingham Malaysia, Semenyih, Malaysia; ^3^ Department of Psychology, Macquarie University, North Ryde, NSW, Australia; ^4^ ARC Centre of Excellence in Cognition and its Disorders, Macquarie University, North Ryde, NSW, Australia; ^5^ Perception in Action Research Centre, Macquarie University, North Ryde, NSW, Australia

**Keywords:** face perception, skin color, perceived health, Asian, culture difference

## Abstract

Facial skin color influences the perceived health and attractiveness of Caucasian faces, and has been proposed as a valid cue to aspects of physiological health. Similar preferences for skin color have previously been found in African participants, while different preferences have been found among mainland Chinese participants. Here, we asked Malaysian Chinese participants (ethnic Chinese living in an Asian country with high levels of exposure to Western culture) to manipulate the skin color of Malaysian Chinese, Caucasian, and African faces to make them “look as healthy as possible.” Participants chose to increase skin yellowness to a greater extent than to increase skin redness to optimize healthy appearance. The slight reduction in skin lightness chosen was not statistically significant after correction for multiple comparisons. While broadly in line with the preferences of Caucasian and African participants from previous studies, this differs from mainland Chinese participants. There may be a role for culture in skin color preferences, though methodological differences mean that further research is necessary to identify the cause of these differences in preferences.

## Introduction

Since the 1990s, evolutionary psychologists have theorized that attractiveness and health judgments serve as a mechanism for identifying a healthy, fertile mate (for a review, see Stephen and Tan, 2015). While some facial cues, such as symmetry and averageness, may be perceived as attractive universally across populations (for reviews, see Rhodes, 2006; Little et al., 2011), other cues, such as body size, appear to vary cross-culturally, either due to cultural or ecological differences between populations (Tovée et al., 2006, 2007).

Facial skin color has been shown to influence perceived attractiveness and health (Stephen et al., 2009b, 2012; Whitehead et al., 2012b; Pezdirc et al., 2017), with increased lightness (represented by the L* dimension in CIELab color space), redness (a*), and yellowness (b*) perceived as healthier. Preference for skin redness may be related to increased blood oxygenation (Stephen et al., 2009a), which serves as an indicator for aerobic fitness and fertility (Armstrong and Welsman, 2001; Charkoudian, 2003; Barelli et al., 2007). Skin luminance is determined by concentration of melanin (Edwards and Duntley, 1939) which is associated with health benefits such as photoprotection and synthesis of Vitamin D (Jablonski and Chaplin, 2000; Kourosh et al., 2010). Skin yellowness is influenced by levels of yellow-red carotenoid pigments in the skin. These carotenoid pigments are obtained from fruit and vegetables in the diet, and then deposited in the top layer of the skin, the *stratum corneum* (Alaluf et al., 2002), and research in Caucasian, Asian, and African populations have linked fruit and vegetable consumption with increased skin yellowness (Stephen et al., 2011; Whitehead et al., 2012a; Tan et al., 2015). Carotenoids are thought to be beneficial for human immunity, visual acuity, and photoprotection of skin (Hughes, 1999; Samimi, 2005; Rao and Rao, 2007; Darvin et al., 2011).

Studies in Caucasian and Asian samples have shown that skin coloration associated with increased intake of fruit and vegetables is perceived as healthy and attractive (Stephen et al., 2011; Whitehead et al., 2012b,c; Lefevre et al., 2013; Lefevre and Perrett, 2015; Pezdirc et al., 2017; Tan et al., 2017), though some studies that did not control for facial expression (Appleton et al., 2018) or color calibration of images and monitors (Jones, 2018) have failed to replicate these results. While similar patterns of preferences for skin lightness, redness, and yellowness coloration have been reported for Caucasian and African samples (Stephen et al., 2009a, 2011; Coetzee et al., 2012), a recent study suggested that mainland Chinese participants show a weaker preference for increased redness and a stronger preference for increased lightness than Caucasian participants and, in contrast to Caucasian and African samples, prefer decreased yellowness (Han et al., 2018).

While the discrepancy between mainland Chinese and other samples may be explained by differences in methodology – Han et al. (2018) used a forced-choice paradigm that did not allow for the elucidation of the amount of color change that was perceived as healthiest – these differences may be attributable to cultural difference in skin color preference. Such cross-cultural differences have been found for other aspects of attractiveness preferences, including preferences for female body size (Swami and Tovée, 2005; Tovée et al., 2006) and male facial masculinity (DeBruine et al., 2010; Brooks et al., 2011), and may relate to cultural differences in cognitive process (Blais et al., 2008; Tan et al., 2012).

Malaysian Chinese, while ethnically Han Chinese, live in a Southeast Asian country that is strongly multicultural (61.7% of the population are ethnic Malay or indigenous, 20.8% Chinese, 6.2% Indian, 0.9% other, and 10.4% noncitizens; CIA, 2018) and influenced by Western culture (86% of movies shown in Malaysian cinemas are Western, 14% local, compared with 56% local movies in China; Epstein, 2011). Previous studies of face perception have found Malaysian Chinese participants to show patterns intermediate between Western and mainland Chinese samples (Tan et al., 2012), and exposure to Western culture has been shown to change individuals’ face recognition strategies (Sangrigoli et al., 2005; Hancock and Rhodes, 2008) and attractiveness preferences (Tovée et al., 2006; Boothroyd et al., 2016). Malaysian Chinese participants have been found to show reduced (though still positive) preference for carotenoid coloration, which contains a large b* component, compared to Western participants in an experimental study (Tan et al., 2017), and to show preferences for lighter and yellower, but not redder, skin in a correlational design (Tan et al., 2018). However, it is not yet known whether Malaysian Chinese show preferences for redness (a*), yellowness (b*), and lightness (L*) in line with Western participants.

Here, we examine Malaysian Chinese participants’ preferences for facial skin color by allowing them to manipulate – separately – facial skin lightness (L*), redness (a*), and yellowness (b*) to optimize the healthy appearance of Asian, Caucasian, and African faces. In line with previous studies (Stephen et al., 2009a,b), we predicted that participants will increase skin redness, yellowness, and luminance to enhance the healthy appearance of faces (Lefevre and Perrett, 2015; Pezdirc et al., 2017; Tan et al., 2018). Previous studies have shown reduced preferences for yellowness and redness, and increased preference for lightness in Asian faces (Tan et al., 2017; Han et al., 2018) but not African faces (Stephen et al., 2011), compared to Caucasian faces, as perceived by own-race observers. However, the influence of color on perceptions of attractiveness has also been shown to be reduced in other-race faces, possibly due to unfamiliarity effects (Stephen et al., 2012). We predict a similar pattern of preferences for Malaysian Chinese participants observing Asian and Caucasian faces, and reduced effects in less familiar African faces.

## Materials and Methods

### Stimuli

Twelve facial photographs of three different ethnicities (four Caucasian, four African, and four East Asian) were obtained from Stephen et al. (2017). These photographs were taken under controlled conditions and color calibrated using Psychomorph (Tiddeman et al., 2001). Hair was held back from the face with a black head band, and participants were asked to pose with a neutral expression while holding a Munsell N5 painted board over their shoulders to obscure clothing.

**Figure 1:**
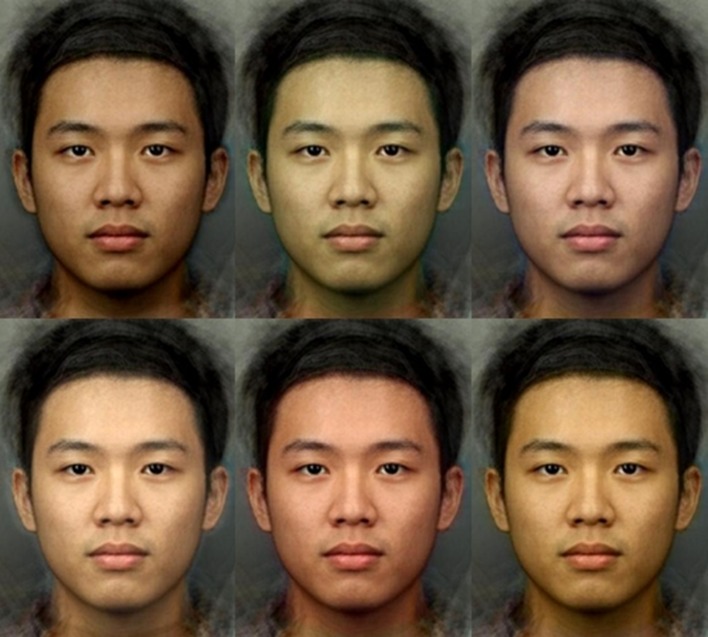
CIELab color transformation of a face with decreased (top) and increased (bottom) lightness (L*, left); redness (a*, middle); and yellowness (b*, right). Presented face is a composite for illustration purposes, but photographs of real individuals were used as the stimuli.

Matlab was used to produce masks with even coloration representing the skin areas of faces, with a Gaussian blur at the edges. One mask was created to represent average face color +8 units of a* (increased redness) and another one with average face color −8 units of a* (decreased redness). Color changes are described using CIE L*a*b* color space, in which colors are described along L* [which takes values between 0 (darkest) and 100 (lightest)], a* [which takes values between −110 (greenest) and 110 (reddest)], and b* [which takes values between −110 (bluest) and 110 (yellowest)], which is designed to reflect the way in which the human visual system processes color information, and is perceptually uniform so that a change of 1 unit in one dimension is perceptually equivalent in magnitude to a change of 1 unit in another dimension (Martinkauppi, 2002). The Euclidean distance (ΔE) between two points in CIE L*a*b* space mirrors the color differences as perceived by human vision (Wyszecki and Stiles, 1982). The facial redness of all the 12 faces used was transformed by the difference in color between each of the pairs of masks, in a series of 13 steps. This produced a series of 13 frames, numbered from 0 to 12, whereby frame 0 had skin redness reduced by 8 units of a*, increasing incrementally so that frame 6 was the original image and frame 12 had skin redness increased by 8 units of a*. Hair, eyes, clothing, and the background were not manipulated. This procedure was repeated for L* (lightness) and b* (yellowness) color axes (Figure 1).

### Participants and Procedure

Forty-four Malaysian Chinese participants (18 males, 26 females; mean age = 22.05, SD = 1.23) were recruited for this study, giving 95% power to detect small to medium effect sizes in the hypothesized main effects and interactions. All participants were students at Universiti Tunku Abdul Rahman.

Stimuli were presented using computers attached to 15” TFT monitors that were color calibrated with a DataColor Spyder3 Pro. Participants were presented with facial images, one image at a time, and were asked to adjust the color of skin portions of the facial images presented to “make the face look as healthy as possible.” By moving the mouse horizontally, the participants cycled through the 13 frames of the transform (same face, different level of color intensity). The participants clicked on the mouse when they felt that the face looked the healthiest.

Each facial image was presented once in each of the three different color dimensions (lightness, redness, yellowness), making a total of 36 trials (12 faces × 3 color dimensions). The location of the midpoint was randomized and the transform looped to obscure the location of the original facial color, and the order of the trials was also randomized in a single block.

## Results

**Figure 2:**
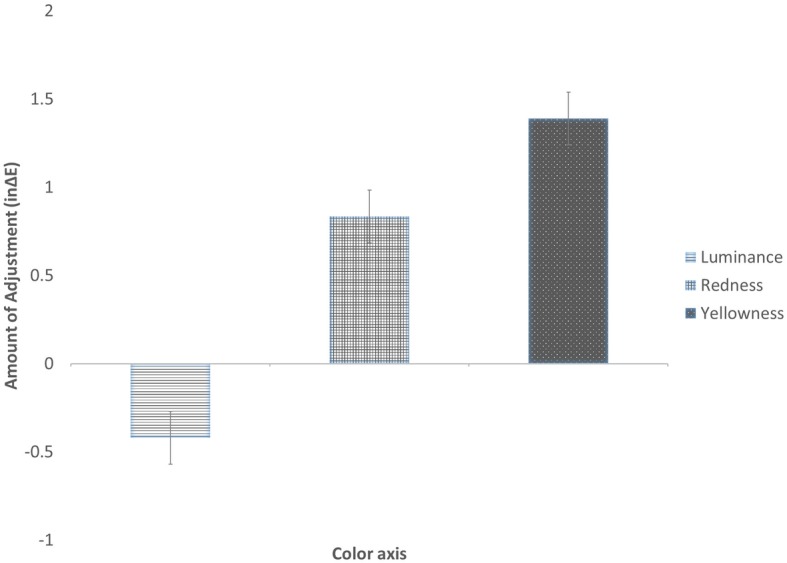
Amount of facial skin color change selected to optimize apparent health of faces.

Mean color changes that were applied to the 12 faces along each color axis were calculated. One-sample *t*-tests showed that participants increased facial yellowness by 1.32 units (SD = 1.28), *t*(43) = 6.85, *p* < 0.001, and facial redness by 0.78 units (SD = 1.09), *t*(43) = 4.72, *p* < 0.001, and decreased facial lightness by 0.37 units (SD = 1.06), *t*(43) = 2.29, *p* = 0.027 (Figure 2), to optimize healthy appearance. Comparing these values to typical values of facial lightness (L*), redness (a*), and yellowness (b*) of the studied populations obtained from previous datasets (Stephen et al., 2012; Tan et al., 2018), they correspond to an increase of 0.62 SD for b*, 0.44 SD for a* and − 0.07 SD for luminance. The result for skin lightness is no longer significant after Bonferroni correction for multiple comparisons.

A 4-way mixed ANOVA was run to examine the differences in the amount of color change applied to the faces of different sexes and ethnicites for all the three color axes, and participants of both genders.

There was a significant main effect for color axes, *F*(2, 84) = 25.91, *p* < 0.001, $\eta_{p}^{2}$ = 0.38. Bonferroni-corrected pairwise comparisons showed a significantly greater increment in facial yellowness than redness (mean difference = 0.55, *p* = 0.037) or luminance (mean difference = 1.81, *p* < 0.001), and greater increment in redness than luminance (mean difference = 1.25, *p* < 0.001).

A significant main effect of ethnicity was found *F*(2, 84) = 41.59.77, *p* < 0.001, $\eta_{p}^{2}$ = 0.50. Caucasian faces received significantly more positive color adjustment as compared to African faces (mean difference = 1.05, *p* < 0.001) and Asian faces (mean difference = 0.36, *p* = 0.004). Asian faces also received more positive adjustment in facial coloration than African faces (mean difference = 0.69, *p* < 0.001).

No significant main effect was found for sex of face, *F*(1, 42) =0.015, *p* = 0.902, $\eta_{p}^{2}$ = 0.000, nor sex of participants, *F*(1, 42) = 2.97, *p* = 0.092, $\eta_{p}^{2}$ = 0.066.

There was a significant interaction for color × gender *F*(2, 84) = 4.357, *p* = 0.016, $\eta_{p}^{2}$ = 0.094. We ran two one-way ANOVAs with repeated measures, and the pairwise comparison showed that, for male participants, there was a significant difference in their adjustment on skin luminance and skin redness (mean difference = −1.88, SE = 0.51, *p* = 0.006) and on skin luminance and skin yellowness (mean difference = −2.50, SE = 0.59, *p* = 0.002). There was no significant difference in adjustment on skin redness and skin yellowness (*p* = 0.35). For female participants, only the adjustment of skin luminance and skin yellowness was significantly different (mean difference = −1.12, SE = 0.26, *p* = 0.001).

There is a significant interaction for ethnic × gender of face × gender, *F*(2, 84) = 4.106, *p* = 0.02, $\eta_{p}^{2}$ = 0.089, which is out of our main research focus. All the other interactions were not significant (*p* > 0.05).

## Discussion

The current study examined Malaysian Chinese participants’ perception of healthy facial skin color. Participants increased the skin yellowness to a greater extent, and increased skin redness to a lesser extent to make faces of three ethnicities look as healthy as possible. The change in skin lightness was not significant after Bonferroni correction. A similar pattern of preferences for skin redness and skin yellowness was observed in previous studies, whereby Caucasian participants significantly increased facial skin yellowness and redness to optimize perceived facial health (Stephen et al., 2009b, 2011; Coetzee et al., 2012; Han et al., 2018). It may be that the observed preference for redder facial skin may be attributable to the appearance of the perfusion of the skin with oxygenated blood, which is associated with physical fitness and increased levels of sex hormones (Armstrong and Welsman, 2001; Charkoudian, 2001; Stephen et al., 2009a). Similarly, increased skin yellowness has been associated with higher levels of deposition of antioxidant carotenoids in the skin, associated with a diet rich in fruits and vegetables (Stephen et al., 2011; Lefevre et al., 2013; Pezdirc et al., 2014, 2017; Tan et al., 2015, 2017). Previous studies also found that preference for skin yellowness was stronger than that of skin redness and skin luminance (Lefevre and Perrett, 2015; Tan et al., 2018), which has been suggested to be related to the antioxidant properties of carotenoids (Paiva and Russell, 1999; Stahl and Sies, 2003), and its protective values to humans’ physical health (Hughes, 2001; Tapiero et al., 2004; Krinsky and Johnson, 2005; Samimi, 2005; Rao and Rao, 2007).

However, it should be noted that Han et al. (2018) failed to find preferences for yellowness in mainland Chinese faces using a two-alternative forced-choice (2AFC) paradigm. Preferences of skin redness for mainland Chinese faces were also not as strong as those observed in the Caucasian sample. The manipulations used by Han et al. (2018), however, were more than double the amount of yellowness increment chosen by participants in the current study, and more than double the amount of carotenoid-induced color change preferred by Malaysian Chinese participants in a previous study (Tan et al., 2017), more than 1.5 SD of the yellowness in an Asian population (Tan et al., 2018), and more than triple the amount of color change preferred by participants in the current study. It may be, therefore, that the high redness and high yellowness images used by Han et al. (2018) were more extreme than looks healthy, and therefore real color preferences may have been obscured.

However, while previous studies have found that Caucasian, African, and mainland Chinese participants choose to increase the lightness of facial skin to optimize healthy appearance (Stephen et al., 2009b; Coetzee et al., 2012; Han et al., 2018), in this study, Malaysian Chinese participants decreased skin lightness, though this preference was no longer significant after Bonferroni correction. While Chinese diaspora culture typically values lighter skin, particularly in women (a common Chinese saying is “a fair skin can hide three facial flaws”; Mak, 2007), this may be offset in the Malaysian context, where ultraviolet radiation from the sun is frequently intense (Kuala Lumpur is less than 400 km from the equator), and increased levels of melanin provides increased protection from sunburn and skin cancer (Jablonski and Chaplin, 2000; Jablonski, 2004).

Previous studies have suggested that skin color changes are more easily detectable in lighter than in darker skinned populations (Coetzee and Perrett, 2014). In the current study, the amount of color adjustment made to optimize the apparent health of faces was greatest for Caucasian faces, followed by Asian, and then African faces, suggesting that skin color may play a greater role in the perception of health in faces from lighter skinned populations.

### Limitations

It should be noted that the current paper allowed participants to manipulate the faces along each color axis separately. However, it may be that the color axes interact such that changes in one color axis affect preferences for color on a different axis. Studies in which all color axes are manipulated simultaneously are required to address this question.

While some discrepancies in skin color preference have been observed across studies conducted at different geographical locations, it cannot be confidently concluded from these data that cultural differences account for the differences between the preferences shown here by Malaysian Chinese participants and participants from Western, African, and mainland Chinese populations. Studies in which methodology is standardized across multiple locations and in which measures of culture are deployed should be conducted to confirm the role of culture, as opposed to methodological differences or ecological differences, in driving the different preferences across populations.

In conclusion, Malaysian Chinese participants show a pattern of facial skin color preference intermediate between that reported in mainland Chinese (Han et al., 2018) and Western (Stephen et al., 2009b) populations, though more similar to the Westerners. While it may be speculated that exposure to Western culture may explain this pattern of results, future studies should standardize methodology across multiple geographical locations, and include measures of culture to confirm this hypothesis.

## Ethics Statement

All subjects gave written informed consent in accordance with the Declaration of Helsinki. The protocol was approved by the Ethics Committee at the University of Nottingham Malaysia Campus.

## Author Contributions

KWT and IS contributed to the conception and design of the study. KWT collected the research data. Both authors performed the statistical analysis, wrote the manuscript, and read and approved the submitted version.

### Conflict of Interest Statement

The authors declare that the research was conducted in the absence of any commercial or financial relationships that could be construed as a potential conflict of interest.
